# mTOR Signaling in Cancer and mTOR Inhibitors in Solid Tumor Targeting Therapy

**DOI:** 10.3390/ijms20030755

**Published:** 2019-02-11

**Authors:** Tian Tian, Xiaoyi Li, Jinhua Zhang

**Affiliations:** College of Life Science and Bioengineering, Beijing Jiaotong University, Beijing 100044, China; ttian@bjtu.edu.cn (T.T.); 15271074@bjtu.edu.cn (X.L.)

**Keywords:** mTOR, PI3K, cancer, inhibitor, therapy

## Abstract

The mammalian or mechanistic target of rapamycin (mTOR) pathway plays a crucial role in regulation of cell survival, metabolism, growth and protein synthesis in response to upstream signals in both normal physiological and pathological conditions, especially in cancer. Aberrant mTOR signaling resulting from genetic alterations from different levels of the signal cascade is commonly observed in various types of cancers. Upon hyperactivation, mTOR signaling promotes cell proliferation and metabolism that contribute to tumor initiation and progression. In addition, mTOR also negatively regulates autophagy via different ways. We discuss mTOR signaling and its key upstream and downstream factors, the specific genetic changes in the mTOR pathway and the inhibitors of mTOR applied as therapeutic strategies in eight solid tumors. Although monotherapy and combination therapy with mTOR inhibitors have been extensively applied in preclinical and clinical trials in various cancer types, innovative therapies with better efficacy and less drug resistance are still in great need, and new biomarkers and deep sequencing technologies will facilitate these mTOR targeting drugs benefit the cancer patients in personalized therapy.

## 1. Introduction

The mammalian or mechanistic target of rapamycin (mTOR) is a serine/threonine kinase that acts through two structurally and functionally distinct protein complexes, mTOR complex 1 (mTORC1) and mTOR complex 2 (mTORC2), to sense and integrate multiple intracellular and environmental signals [1,2]. mTOR signaling is generally involved in regulating cell survival, cell growth, cell metabolism, protein synthesis and autophagy, as well as homeostasis [3]. The pathological relevance of dysregulation of mTOR signal is illustrated in many human diseases, especially the multitude of different human cancers. As reported, mTOR is aberrantly overactivated in more than 70% of cancers [4]. Over the past few years, it has been extensively demonstrated in animal models and clinical patients of cancer that mTOR dysfunction contributes to tumorigenesis [5].

Since the mTOR pathway regulates many basic biological and physiological processes such as cell proliferation, survival and autophagy, it is logical that components in the mTOR pathway are among the most frequently mutated genes in cancers [6]. The regulation of mTOR pathway is also influenced by its positive and negative regulators that have cross talk with mTOR, such as the phosphoinositide 3-kinase (PI3K)/Akt, mitogen activated protein kinase (MAPK), vascular endothelial growth factor (VEGF), nuclear factor-κB (NF-κB), and p53 etc., which comprise a much more complicated signaling cascade [7].

Several types of mTOR inhibitors such as rapamycin, its rapalogs and mTORC1/2 kinase inhibitors have been examined in various cancer models, including breast cancer, lung cancer, gastric carcinoma, colorectal cancer, prostate cancer, head and neck cancer, gynecologic cancer, glioblastoma, lymphoma, urinary bladder cancer, renal cancer and medulloblastoma, etc. However, the effects of mTOR inhibitors utilized as monotherapy in cancer are sometimes dampened by several resistance mechanisms [8]. Combined therapies with mTOR inhibitors and other pathway inhibitors or conventional therapies are under investigation in preclinical and clinical trials in different tumor types. Hence, novel therapeutic strategies based on mTOR inhibition still need to be developed.

## 2. mTOR (The mammalian or mechanistic target of rapamycin) Signaling in Cancer

### 2.1. mTORC1 and mTORC2

**Figure 1:**
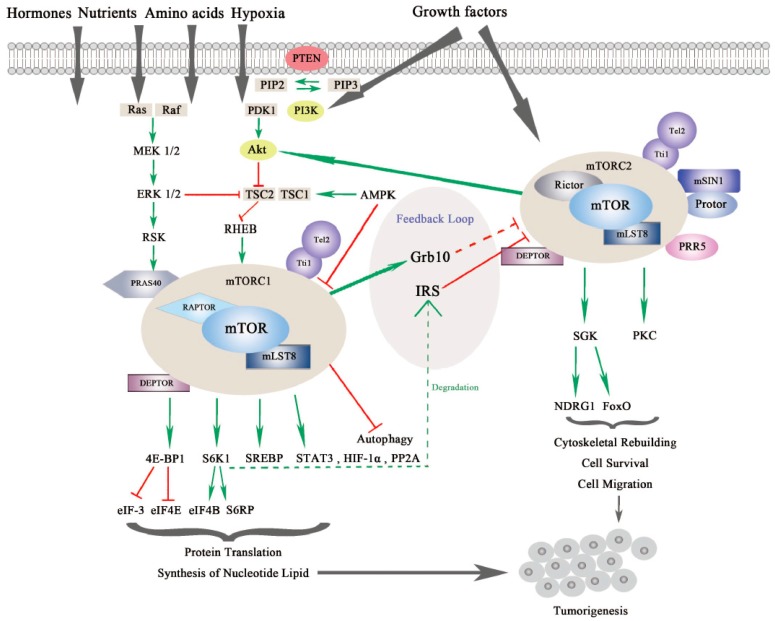
The mammalian or mechanistic target of rapamycin (mTOR) complexes and signaling pathway of mTORC1 and mTORC2. mTORC1 is responsive to nutrients, hormones, amino acids, hypoxia and growth factors, while mTORC2 responds to growth factors. mTORC1 and mTORC2 share common subunits of mTOR kinase, mLST8, DEPTOR (DEP domain-containing mTOR-interacting protein), Tel 2 and Tti 1. mTORC1 additionally binds with RAPTOR (Regulatory-associated protein of mTOR) and PRAS40 (Proline-rich substrate of 40 kDa), and mTORC2 combines with RICTOR and mSIN1 (Mammalian stress-activated protein kinase interacting protein 1) as well as Protor and PRR5 (Proline-rich protein 5). mTORC1 is regulated by PI3K/Akt (Phosphoinositide 3-kinase/serine-threonine protein kinase) and Ras-MAPK (Mitogen activated protein kinase) signaling pathways. mTORC1 regulates protein translation and synthesis of nucleotide lipid via 4E-BP1 and S6K1 and downstream effectors. mTORC1 also activates STAT3 (Signal transducer and activator of transcription), HIF-1α (Hypoxia-inducible factor 1α) and PP2A (Protein phosphatase 2A) in tumorigenesis. mTORC2 regulates SGK (Serum glucose kinase) and PKC (Protein kinase C) to promote cell survival, cytoskeleton reorganization and cell migration. mTORC2 is negatively modulated by mTORC1 via different feedback loops mediated by IRS (insulin receptor substrate) or Grb10. mTORC1 and mTORC2 can both contribute to turmorigenesis through different mechanisms [7,11].

mTOR is a serine/threonine kinase, which is attributed to the phosphoinositide 3-kinase related protein kinase (PIKK) super family, and was first discovered from a genetic screening for rapamycin-resistant mutations in yeast *Saccharomyces cerevisiase* [9,10]. In mammalian cells, mTOR mainly acts through its two evolutionarily conserved complexes, mTORC1 and mTORC2, which share some common subunits, such as the mTOR kinase, the mammalian lethal with SEC13 protein 8 (mLST8), dishevelled, EGL-10 and pleckstrin (DEP) domain-containing mTOR-interacting protein (DEPTOR), telomere maintenance 2 (Tel2) and Tel2-interacting protein 1(Tti1) complex as shown in Figure 1.

mTORC1 and mTORC2 are different in the aspects of rapamycin sensitivity, specific binding components, subcellular localization, downstream substrates, and regulation [12]. mTORC1 is sensitive to rapamycin whereas mTORC2 is comparatively resistant to rapamycin [13]. In addition to the common binding subunits, mTORC1 and mTORC2 respectively harbor distinct components that contribute to the specificity of substrates, different subcellular localization, and specific regulation. mTORC1 also contains the regulatory-associated protein of mTOR (RAPTOR), which is a significant scaffolding protein in the mTORC1 assembly and its stability and regulation, and proline-rich substrate of 40 kDa (PRAS40) is a negative regulator of mTORC1 by releasing mTORC1 inhibition upon the activation of growth factors [14,15]. mTORC2 uniquely contains rapamycin-insensitive companion of mTOR (RICTOR) and the mammalian stress-activated protein kinase interacting protein 1 (mSIN1), both of which can mutually affect their protein levels and stabilize each other. Previous research has demonstrated that RICTOR is a scaffolding protein essential for the assembly, stability, substrate recognition, and subcellular localization activation of mTORC2. In addition, mSIN1, which is essential for plasma membrane localization of mTORC2, negatively regulates mTORC2 kinase activity [16,17]. Newly discovered interactors include Protein observed with RICTOR 1/2 (Protor-1/2), which are required for mTORC2 assembly and catalytic process, and Proline-Rich Protein (PRR) 5, which is necessary for mTOR activity and mTOR–RICTOR binding [18,19].

mTORC1 and mTORC2 have differing subcellular localization binding with their own respective, specific subunits, which also determine their distinct functions and independent regulations. mTORC1 is associated with endosomal and lysosomal membranes, where it interacts with its effectors. mTORC2 is affiliated with the plasma membrane, as well as ribosomal membranes, where it binds with its key substracts, AGC family kinases (subgroup of Ser/Thr protein kinases named after 3 representative families, the cAMP-dependent protein kinase (PKA), the cGMP-dependent protein kinase (PKG) and the protein kinase C (PKC) families), such as serum glucose kinase (SGK) isoforms and protein kinase C (PKC), which are essential for mTORC2 activation [20]. Both mTORC1 and mTORC2 play significant and differing roles in a variety of intracellular processes. They are regulated by various endogenous and exogenous stimuli, such as nutrients, growth factors, energy, hormones and hypoxia, and they can also affect glucose metabolism through different physiological mechanisms [1,21,22,23]. Generally, mTORC1 can phosphorylate its downstream effectors, such as eukaryotic translation initiation factor 4E binding protein 1 (4EBP1), S6 kinase (S6K), and sterol regulatory element-binding protein (SREBP), to motivate protein translation, synthesis of nucleotides and lipids, biogenesis of lysosomes, and to suppress the process of autophagy [24]. On the other hand, mTORC2 is more sensitive to extracellular growth factors though the molecular mechanism remains to be elucidated [25]. Upon activation, mTORC2 phosphorylates its downstream targets SGK and PKC, as mentioned previously, to intensify the signaling cascade [26]. mTORC2 mainly increases cytoskeletal rebuilding and cell migration, inhibits apoptosis and affects metabolism [27] (as shown in Figure 1).

### 2.2. Signaling of mTORC1

The mTOR signaling pathway is crucial in cell growth, proliferation and metabolism. mTORC1 is regulated by several signaling pathways including the PI3K/Akt pathway, the Ras-MAPK pathway, and some other intracellular factors (see Figure 1).

Activation of mTORC1 is primarily dependent on the PI3K/AKT pathway to respond to oncogenic growth factors or insulin [28]. Even though the second messenger phosphatidylinositol (3,4,5)-triphosphate (PIP3) binds and activates mTORC2 directly, mTORC1 can also be indirectly activated by PI3K through Akt. Akt is activated by phosphorylation at Ser473 by mTORC2 and at Thr308 by another serine-threonie kinase PDK1 (Phosphoinositide-dependent Kinase 1). Then, phosphorylation of tuberous sclerosis complex 2 (TSC2) by active Akt results in blockage of TSC2 and TSC1 combination [29,30,31]. The activator of mTORC1, Ras homolog enriched in brain (RHEB), which is negatively regulated by TSC1/2, is released by TSC to allow the activation of mTORC1 in lysosomes [32]. In addition, AKT can activate mTORC1 by phosphorylating and dissociating the inhibitor PRAS40 from RAPTOR independent of TSC1/2 [33].

Moreover, TSC2 can also be phosphorylated by extracellular signal-regulated kinases (ERKs) and ribosomal protein S6 kinase (RSK) from the Ras-MAPK signaling pathway, which results in inhibiting TSC1/2 and promoting RHEB-mediated mTORC1 activation. In addition, similar to AKT, PRAS40 can also be phosphorylated by RSK to release RAPTOR and activate mTORC1 [34,35,36].

mTORC1 is also responsive to fluctuations of cellular factors such as DNA damage, intracellular adenosine triphosphate (ATP), glucose, amino acids, and oxygen. Several signaling pathways that are responsive to DNA damage suppress mTORC1 via p53 target genes, leading to TSC2 activation: for example, 5′-AMP activated protein kinase β (AMPKβ) and phosphatase and tensin homolog on chromosome 10 (PTEN) [37]. Upon energy exhaustion, AMP kinase (AMPK), which is activated by low ATP/high AMP levels, promotes TSC1/2 complex formation and phosphorylates RAPTOR, leading to indirect inhibition of mTORC1 [38]. This outcome also implies that in a situation of energy shortage, AMP accumulation will cover the growth factor signals and suppress cellular replication. Through a sensing signal cascade of amino acids, mTORC1 can be positively regulated by amino acids, activation of which motivates the Rag complex to combine with RAPTOR. Along with this process, mTORC1 is recruited to the lysosomal surface [39,40]. Rag-GTPase, which is associated with RAPTOR and localizes mTORC1 to lysosomal membranes, is especially activated by arginine in lysosomes or by leucine in the cytoplasm [41,42,43,44].

Once activated, mTORC1 will transfer the signal to downstream effectors, such as 4EBP1 and S6K1, both of which are essential modulators of cap-dependent and cap-independent translation. After phosphorylation of 4EBP1 and S6K1 by mTORC1, the binding partners, eukaryotic initiation factor (eIF)-4E and eukaryotic initiation factor-3 (eIF-3), will be respectively liberated, facilitating initiating complex formation for translation and intensifying ribosome genesis [45]. In the following signal cascade, eIF-4E will form the eIF-4F complex and increase protein translation, which is significant for the G1-S phase transition. Upon low mTORC1 activity, 4E-BP1 is dephosphorylated, and protein translation is inhibited [46]. On the other side, eIF-4B and S6 ribosomal protein (S6RP) are phosphorylated by S6K1, which initiates protein translation and continues translation elongation [47,48]. Actually, mTORC1-related signals seem to prefer to affect the translation of oncogenic proteins involved in protein synthesis, invasion and metastasis [49]. Moreover, mTORC1 also regulates some other proteins such as hypoxia-inducible factor 1α (HIF-1α), protein phosphatase 2A (PP2A), glycogen synthase, and signal transducer and activator of transcription (STAT) 3, through which mTORC1 promotes biosynthesis of proteins, lipids and nucleotides in aberrant cells, tissue and organism growth in cancer [2,50,51,52,53,54].

In brief, mTORC1 activation induces cap-dependent translation that leads to increases in cell size and proliferation, which are two typical characteristics of cancer [55,56].

### 2.3. Signaling of mTORC2

Although the regulatory mechanism of mTORC1 is well depicted, the regulators of mTORC2 are much less characterized. This is partly due to the difficulties in teasing apart the functional differences between mTORC1 and mTORC2 [13]. As we mentioned previously, through mSIN1, mTORC2 localizes at the plasma membrane where it binds with its substrates Akt, SGK and PKC. Notably, the localization of mTORC2 is significant for its regulation [16] (see Figure 1).

First, mSIN1 regulates mTORC2 depending on different mechanisms. mTORC2-Akt signaling can be sustained by a positive feedback loop from mSIN1 phosphorylation of Akt, whereas mSIN 1 phosphorylation by S6K1 at the same site suppresses mTORC2 activity [57,58,59]. On the other hand, recent research found that mSIN1 can also combine with Rb in the cytoplasm, which results in the inhibition of mTORC2 complex formation and Akt signaling [60].

Likewise, mTORC2 is regulated by PI3K/Akt, as well as by mTORC1 itself. PI3K activates mTORC2 to bind to ribosomes both in normal physiological and pathological conditions, such as cancer [61]. Akt, which is commonly found to be hyperactive in cancers, is an important substrate of mTORC2. Akt aggregates signals from PI3K/mTORC2 and PI3K/PDK1 to accelerate cell proliferation. Localization of Akt to the plasma membrane is regulated by PIP3, which is similar to mTORC2. Akt also activates mTORC1 signaling in addition to mTORC2, leading to a more complicated signal network [29]. In addition, mTORC2 is negatively modulated by mTORC1 via feedback loops. For example, the S6K1 promotes insulin receptor substrate (IRS) 1/2 degradation resulting in inhibition of mTORC2 and the PI3K/Akt pathway. Another feedback mechanism is through growth factor receptor-bound protein 10 (Grb10), which is positively modulated by mTORC1 [62,63,64].

For downstream effectors, serum and glucocorticoid kinase (SGK) and protein kinase C (PKC) are two key phosphorylation substrates of mTORC2. SGK substrates include N-myc downstream-regulated gene 1 protein (NDRG1) and Forkhead box family transcription factors (FoxO), which promote cell survival under oxygen or nutrient depletion conditions or in response to PI3K inhibition [65,66]. Through phosphorylation of different PKC family members, mTORC2 is reported to regulate cytoskeleton reorganization and cell movements involved in tumorigenesis [17,25,67,68] (See Figure 1).

### 2.4. mTOR Signaling in Cancer

Since mTOR signaling regulates fundamental activities including cell cycle, proliferation, growth, and survival, as well as protein synthesis and glucose metabolism, there is no doubt that mTOR has a close association with cancer. As reported, mTOR signaling is enhanced in various types of cancers. Data in solid tumors demonstrated that the mTOR signal is dysregulated in almost 30% of cancers and is one of the most frequently affected cascades in human cancers [69].

Activation of mTOR signaling in cancer mainly depends on three different levels of mechanisms: first, mutations in the mTOR gene lead to a constitutively hyperactive mTOR signaling cascade; second, mutations in the components of mTORC1 and mTORC2 result in activation of mTOR signaling; and lastly but most importantly, aberrant mTOR signaling can also result from mutations in upstream genes, that is, loss-of- function mutations in suppressor genes and gain-of-function mutations in oncogenes [7]. We discuss these mechanisms in the following text.

Mutation of mTOR, which is the core gene of the mTOR signaling and encodes the kinase, will directly lead to hyperactivation of mTOR signaling. A study utilizing public tumor genome sequencing data in 2014 reported that 33 mTOR mutations were found to contribute to the hyperactivation of mTOR signaling in various cancer types. Most of these mutations assemble in six different regions of the c-terminal region of mTOR in several cancer types, and one is specifically abundant in kidney cancer, all of which maintain the sensitivity to mTOR inhibition by pharmacological therapies [70].

Moreover, genetic aberrations in components of mTOR complexes are reported to have a close relationship with cancer. RICTOR, a component of mTORC2, was found to be amplified in beast cancer, non-small cell lung cancer (NSCLC), and particularly in squamous cell lung carcinoma (SQCLC), in which RICTOR amplification is significantly related to poor prognosis and short survival [71,72,73]. Overexpression of RICTOR was also observed in gliomas with high Akt activity in nearly 70% of patients and HER2 (human epidermal growth factor receptor-2)-positive breast cancers, leading to Akt hyperactivity and tumor aggravation [72,74].

Except for the above, mTOR signaling hyper-activation can commonly result from mutations of upstream genes including oncogenes and tumor suppressor genes [75]. The PI3K signaling pathway, which is upstream of both mTOR complexes, often has various kinds of mutations of its components in cancer, such as mutation and amplification of Akt and of PIK3CA and amplification of growth factor receptors, Epidermal Growth Factor Receptor (EGFR) and insulin growth factor receptor (IGFR) [76,77,78]. Since PI3K and RAS are two parallel pathways, amplification of growth factor receptors that are upstream of either signal can also result in abnormal signal transduction on both mTOR complexes [6]. Furthermore, loss of functions in tumor suppressor genes, such as PTEN, p53, TSC1/TSC2 and Serine Threonine Kinase 11 (STK11), all contribute to mTOR activation in the pathological state of cancer [79]. PTEN, which is the second most frequently mutated gene after p53 in human cancer, can be downregulated through mutation, methylation, protein instability and intracellular localization [80]. Aberrations in the PTEN genes also influence cancer cells in myeloma, breast cancer and endometrium cancer, which are sensitive to mTOR inhibitors [81,82,83,84]. Inactivation of TSC1 or TSC2, which are negative regulators of mTORC1, is responsible for Tuberous Sclerosis and leads to benign tumor genesis. This also demonstrates that mTORC1 serves as a potent driver of cell proliferation. Mutations of TSC1 and TSC2 are reported in bladder cancer, urothelial carcinoma, clear cell renal carcinoma and well-differentiated pancreatic neuroendocrine tumors [85,86,87]. Actually, mutations in TSC1, TSC2 and mTOR are much less frequent than those in components that are higher upstream in the signaling pathway.

mTOR signaling mainly regulates cell proliferation and metabolism involved in tumor initiation and progression. As reported, at the level of 4E-BP1/eIF-4E, dysregulation of protein synthesis downstream of mTORC1 play a central role in tumorigenesis. eIF-4E promotes the translation of specific pro-oncogenic proteins that regulate cell survival, cell cycle progression, angiogenesis, energy metabolism, and metastasis. Besides, mTOR activation also leads to increased ribosome biogenesis, providing machinery to maintain high levels of cell growth [1]. In cancer cells, metabolism seems to reprogram to sustain the demands of rapid cell growth. mTOR complex is recently depicted as a nutrient sensor in metabolism of cancer, especially on glucose and amino acid, nucleotide, fatty acid and lipid, growth factors and other stresses. Nutrient sensing mainly activates mTORC1 and the metabolic changes in cancer cells sustain mTOC1 activation in turn [2,22,23,88]. In glucose metabolism, mTORC1 can enhance the translation of two key transcription factors, hypoxia inducible factor (HIF)-1α and Myc, which drive expression of a variety of glycolytic enzymes to regulate glycolysis [89,90,91]. mTORC2 can also increase glucose metabolism through its downstream effector AKT [92]. For lipid synthesis, mTORC1 activates the critical transcription factor sterol regulatory element-binding protein 1 (SRE-BP1) driving gene transcription in lipid synthesis via Akt activation and phosphorylation of Lipin1 and S6K1 [93,94]. The increased levels of SRE-BP mRNA and protein are associated with mTORC1 upregulation in human breast cancer tissues [95]. In addition, purine and pyrimidine synthesis, which is significant for cancer cell DNA replication, can also be promoted by mTORC1 via S6K1 phosphorylation [96,97].

Moreover, mTOR is involved in the regulation of autophagy, a process that degrades and recycles cytosolic components in response to a shortage of nutrients and energy. Autophagy is commonly regarded as an inhibition process against tumorigenesis, and blockage of autophagy contributes to cancer initiation [98]. However, some conflicting research results have demonstrated that autophagy may play a dual role in cancer development under specific conditions: for example, it is dependent on different P53 status in pancreatic cancer [99,100,101]. mTORC1 is reported to inactivate UNC-5-like autophagy-activating kinase 1 (ULK1) by phosphorylation resulting in failure to form ULK1-ATG13-FIP200 complex, which is required for autophagy initiation [102,103,104], while mTORC2 can inhibit autophagy indirectly by activating mTORC1. mTORC1 also regulates autophagy at the transcription level by modulating a key transcription factor, Transcription Factor EB (TFEB), for genes in lysosomes and autophagy [105]. Moreover, mTORC1 is likely to affect autophagy through some other ways such as the death-associated protein 1 (DAP1) which suppresses autophagy, WD repeat domain phophoinositide-interacting protein 2 (WIPI2) and a mammalian ortholog of Atg18 [23].

## 3. mTOR Inhibitors in Therapies of Different Types of Cancer

As stated above, the mTOR signaling pathway plays a central role in cancer initiation and progression, and is the second most frequently altered pathway after the p53 pathway in human cancers [106]. Therapies utilizing mTOR inhibitors have been developed to reduce the high mTOR signaling levels in various cancer types.

Rapamycin, which lead to mTOR discovery of mTOR in the target screening, is the original inhibitor of mTOR. Rapamycin binds to FK506 Binding Protein 12 (FKBP12), resulting in the unbinding RAPTOR from mTORC1. In addition, the downstream effect is inactivation of S6K1 and 4E-BP1 by inhibiting phosphorylation, which leads to a decrease in protein synthesis and cell cycle arrest in the G1 phase [107]. Rapamycin also negatively regulates VEGF, platelet-derived growth factor (PDGF), basic fibroblast growth factor (bFGF) and so on, which are transcriptional targets of hypoxia-inducible factor 1α (HIF-1α) and contribute to vascular development and cancer progression [108]. Moreover, rapamycin can act indirectly on mTORC2 also by binding FKBP12, leading to dissociation of RICTOR from mTOR, thus decreasing the levels of mTORC2 and possibly in a specific cell type [13,109]. Due to poor solubility and undeterminate kinetic and pharmacological properties of rapamycin, a series of allosteric mTOR inhibitors (named rapalogs) have been developed to achieve better efficacy in patients [110]. Four rapalogs of rapamycin: temsirolimus (by intravenous administration), everolimus and ridaforolimus (by oral administration) and ABI-009 (nanoparticle albumin-bound-rapamycin) have been applied in monotherapy or combination therapies in a variety of cancer types in different phases of clinical trials [111]. Apart from rapalogs, ATP competitive inhibitors, such as vistusertib (AZD2014), AZD8055, CC-223 and OSI027 that suppress mTORC1 and mTOR2 kinase simultaneously, and PI3K/mTOR dual inhibitors might result in improved anticancer effect in preclinical and clinical studies [112]. Several potential biomarkers, including PIK3CA and PTEN mutation status, AKT activity, and other members of the mTOR pathway, have also been explored according to preclinical results and clinical data.

In the following parts of our review, we focus on the alterations of mTOR signaling in eight different types of solid tumors and applications of various mTOR inhibitors in therapeutic strategies in these specific tumors.

### 3.1. Lung Cancer

In non-small cell carcinomas (NSCLC), PI3K pathway activation is found in 50–70% of patients with AKT phosphorylation [113]. Mutations in EGFR, Kirsten rat sarcoma viral oncogene (KRAS), PI3K, amplification of PIK3CA and loss of PTEN can lead to PI3K pathway activation [114]. As reported by The Cancer Genome Atlas (TCGA) Research Group, alterations in the PI3K/Akt pathway, which is upstream of mTOR signaling, were detected in 47% of squamous cancers (including *PIK3CA* alterations in 16%, *PTEN* alterations in 15%, *AKT3* alterations in 16%, *AKT2* alterations in 4% and *AKT1* alterations < 1% of the total samples) [114]. Actually, genomic amplification is much more frequent than somatic mutations in *PI3KCA* in lung cancers. In addition, *PI3KCA* was found to have copy number amplifications in 33% of squamous cell lung carcinomas, which occurred independently of the *PI3KCA* gene mutation, demonstrating that each event is probably sufficient to initiate tumorigenesis. Besides, in a report of 51 Japanese small cell lung cancinoma (SCLC) patients, 36% of the tumors had genetic mutations related with mTOR pathway [115]. Phophorylated mTOR is demonstrated to contribute to SCLC progression [116].

Although some reports indicated that expression of mTOR/phosphorylated-mTOR (p-mTOR) has no significant association with prognosis in NSCLC patients [117], mTOR inhibitors including everolimus, temsirolimus, and ridaforolimus have been extensively applied in NSCLC patients in clinical trials. Although both everolimus and ridaforolimus demonstrated promise in phase I studies, neither of them achieved such promising results in phase II studies in NSCLC patients due to toxicity of these traditional mTOR inhibitors [118,119,120]. Everolimus either combined with chemotherapy (CT) or radiotherapy also showed non-significant results in NSCLC patients [121,122]. A phase II study in advanced NSCLC patients treated with chemotherapy (CT) or CT and EGFR inhibitors demonstrated that everolimus at a dose of 10 mg/day achieved a response rate of 4.7% and a disease control rate of 47.1% [123]. Another phase II clinical trial of everolimus (5 mg/day) combined with the EGFR inhibitor gefitinib (250 mg/day) in 62 advanced NSCLC patients did not indicate a definite result because the partial response rate did not meet the threshold to continue further investigation [124]. Temsirolimus is reported to suppress cell proliferation in NSCLC cell lines relying on different doses [125]. A phase I clinical trial of temsirolimus confirmed a partial response rate in one patient with NSCLC out of 63 patients of various types of advanced cancer [126]. In a phase II study, 35% of NSCLC patients (*n* = 52) benefited from temsirolimus, among which 8% patients had confirmed PR and 27% had a stable disease [127]. On the other hand, both everolimus and temsirolimus have some adverse events (AEs), such as fatigue, dyspnea, stomatitis, mucositis, asthenia, nausea and mucositis, and combination therapies with other inhibitors, radiotherapy or chemotherapy are still under investigation. Sirolimus, which is an allosteric inhibitor of mTORC1, was demonstrated to possibly inhibit the NSCLC cell proliferation in a preclinical study [128]. Clinical trials are still under way in phase I or II of sirolimus combined with other therapies in patients with NSCLC harboring specific gene mutations [121]. Reports on mTOR inhibitors in SCLC are relatively rare, and temsirolimus was shown to fail to benefit SCLC patients [129].

### 3.2. Gastric Cancer (GC)

Researches demonstrated that PIK3CA, PIK3CB, AKT1 and mTOR are overexpressed in GC cell lines, and mTOR pathway is active in almost 60% of gastric cancer patients [130]. *PIK3CA* is reported to be commonly mutated and amplified at frequencies of around 18% and 5%, respectively [131]. Three mutation hotspots that exist in almost 80% of *PIK3CA* mutations are E545K (exon 9), E542K (exon 9) and H1047R (exon 20) [132]. As reported, *PIK3CA* mutation frequency in gastric cancer is associated with cancer stage and Epstein–Barr virus (EBV) infection [131,133]. *PTEN*, which is a key inhibitor of the PI3K pathway, is a significant tumor suppressor gene. According to the TCGA database of gastric cancer, deletion, mutation and amplification of *PTEN* each occur in 0.3%, 3.1% and 4% of cases, respectively. The alteration frequency of *PIK3CA* and *PTEN* varies significantly in different populations: for example, between Asian and Caucasian GC patients, the rate is 7% compared to 15% for *PIK3CA* mutations, 21% compared to 4% for *PTEN* deletion, and 47% compared to 78% for *PTEN* loss, respectively [134]. Another research found 19% *PTEN* mutations in GC patients in a Chinese population, including missense, nonsense, deletion, and mutations in intron 6 [135]. PTEN tends to be mutated more frequently in advanced stage or less differentiated GC [136]. Despite AKT overexpression in 74% of GC patients examined by immunochemistry, the genetic alterations in AKT are very few at approximately 1% to 3% in GC [137,138]. Although the exact genomic changes that occur in mTOR signaling downstream of PI3K/Akt are not well clarified, it is reported that phosphorylated-mTOR overexpression is related to some clinicopathological features and poor prognosis in GC patients alone or combined with TSC1 downregulation [139,140]. In an Eastern Chinese population, mTORC1 polymorphisms contribute to the risk of GC [141]. Moreover, an immunohistochemical study via GC tissue microarray demonstrated that aberrant S6K1 expression may lead to cancer initiation, invasion and metastasis of GC [142].

The mTOR inhibitors have also been utilized in preclinical studies and clinical trials of GC. Everolimus and sirolimus showed obvious G1 cell cycle arrest effects and suppressed proliferation in gastric cancer cell lines [143,144]. Rapamycin was responded well in cancer cells harboring PIK3CA and/or PTEN mutations (*P* = 0.0123) in a preclinical study, and inhibited tumor volume and microvasculature growth when was applied in a mouse xenograft model [145,146]. Temsirolimus demonstrated a favorable toxicity profile, pharmacokinetics features, and cancer resistant efficacy in a phase I trial in advanced cancer including GC and is continuing to a phase II trial [126]. Everolimus showed a good disease control rate (DCR) (56%), median progression-free survival (PFS; 2.7 months) and overall survival (OS; 10.1 months) in advanced GC patients (*n* = 53) in phase II trails [147]. And biomarkers exploration has also been executed in a phase II study of everolimus in advanced gastric cancer patients, and pS6 (Ser240/4) was found to be a potential predictive marker [148]. Although some side effects of everolimus (stomatitis, anorexia, fatigue, rash, nausea, peripheral edema, diarrhea and pruritus) existed and improvements of the overall survival and primary endpoint were not obvious, the PFS for six months and safety were significant in previously treated GC patients in phase III trials, which also made everolimus the only drug to progress to phase III tirals for advanced GC treatments [149,150]. Ridaforolimus, also an analog of rapamycin, demonstrated good antitumor effects during preclinical and phase Ib clinical trials combined with capecitabine [151]. mTORC1/2 kinase inhibitors other than rapalogs, such as PP242, AZD 2014, AZD8055, and OSI-027 have also attracted interests due to their competition with ATP in mTOR kinase activity. PP242 showed outstanding antiproliferative and antiangiogenesis capabilities in GC cell lines, while there are no future reports of other inhibitors on GC therapies so far and most of these inhibitors are still in phase I trials [138,152].

### 3.3. Colorectal Cancer (CRC)

The PI3K/Akt pathway is genetically altered in many CRC cell lines [153]. Mutations of PI3K and PTEN are dominant among those alterations in CRC patients. As reported, approximately 15% of metastatic CRC patients carried *PI3K3CA* mutations, and loss of PTEN was found in 20% to 40% of CRC patients [154,155]. In addition, PI3K subunit p85α and AKT1/2 were overexpressed, particularly in advanced tumor stages, and the phosphorylation level of mTOR and S6K1 was increased in CRC [156]. Mutation of the p53 gene or deletion of the 17p chromosome is significant for tumor initiation, especially from adenoma to carcinoma in CRC [157,158]. p53 inhibits mTOR activity via AMPK-β1 and TSC2 in CRC cell lines. p53 also regulates the mTOR pathway by a target gene, DNA damage and development 1 (REDD1), which is essential for hypoxia activation of TSC1/2 and modulated by oxidative stress [159,160]. Previous immunohistochemical studies demonstrated that mTORC1 signaling was involved in tumorigenesis at an early stage and contributed to progression from normal cells to a neoplastic state in human colorectal adenoma and cancers [161]. mTORC1 and mTORC2 both overexpress and play significant roles in CRC.

As for clinical trials of mTOR inhibitors, neither everolimus nor temsirolimus showed satisfactory effects as monotherapies in treating metastatic CRC in several clinical trials. The effects of temsirolimus were limited, especially in metastatic CRC patients with KRAS mutations [162,163]. Partial suppression of the mTOR signaling pathway by rapamycin and rapalogs was found to be attributed to 4E-BP1 kinase, which led to resistance in CRC [164]. Combination treatments of rapalogs and other drugs have exhibited potential in CRC therapies. For example, combination of the VEGF inhibitor bevacizumab and an mTOR inhibitor achieved fewer adverse effects and prolonged stable disease in metastatic CRC patients [165]. Sorafenib was reported to improve the efficacy of rapamycin in CRC patients harboring *K-RAS* and *PIK3CA* mutations [166]. Everolimus together with octreotide LAR (long-acting release) achieved an obviously prolonged PFS in advanced colorectal neuroendocrine cancers in a phase III study, while the combination of everlimus and irinotecan was well tolerated in a phase I study in mCRC patients [167,168]. And everolimus and tivozanib, which inhibits angiogenesis, demonstrated a 50% disease control in a phase II trial [165]. The combined therapies with mitogen-activated protein kinase kinase (MEK) inhibitors and mTOR inhibitors also attracted more attention because these treatments can overcome the resistance to MEK suppression in CRC [169]. Moreover, dual PI3K/mTOR inhibitors have a reduced possibility to induce drug resistance than rapalogs, and mTOR kinase inhibitors can suppress mTORC1 and mTORC2 simultaneously; thus, these drugs are introduced as the second generation of mTOR inhibitor drugs in preclinical and clinical trials [170]. For instance, NVP-BEZ235, a dual inhibitor of PI3K and mTOR signaling, inhibited tumor growth in a genetically engineered mouse model of sporadic CRC [171]. mTORC1/2 inhibitors OSI-027 showed obvious antitumor activity in several human xenograft models with various histologies [172,173]. In another study of human colon cancer cell line xenograft, both the ATP competitive mTOR inhibitor PP242 and dual inhibitor of PI3K and mTOR NVP-BEZ235 significantly suppressed the xenograft growth, and they achieved better efficacy combined with a MEK inhibitor, implying a prosperous future for second generation mTOR inhibitors in combination therapies for CRC [174].

### 3.4. Renal Cancer (RCC)

RCC is regarded as one of the most lethal cancers because of the rare available therapies and lack of proper diagnosis biomarkers at early stages. RCC is mainly classified as clear cell renal cell carcinoma (ccRCC, 85%), papillary renal cell carcinoma (PRCC, 0–15%), chromophobe renal cell carcinoma (chRCC, 5%) and collecting duct carcinoma and medullary carcinoma (1%).

Generally, mTOR signaling regulates cell metabolism, and RCC is also a cancer of metabolism dysregulation [175]. Data from TCGA on a ccRCC study in 2013 demonstrated genetic alterations in components of each level of the PI3K/Akt signaling pathway cascade (PIK3CA, PIK3R1, PIK3R2, PTEN, PDPK1, AKT1, AKT2, AKT3, FOXO1, FOXO3, MTOR, RICTOR, TSC1, TSC2, RHEB, AKT1S1, and PRTOR), mainly including *GNB2L1* amplification (6%), *PI3KCA* amplifications or mutations (5%), *PTEN* deletions or mutations (5%) or *MTOR* mutations (6%). Clustered *MTOR* mutations, as well as mutations in *AKT1*, *AKT3* and *RHEB*, contributed to PI3K/Akt and mTOR hyperactivation in ccRCC [70,176,177]. In addition, the cross talk between VHL/HIF and the PI3K/Akt pathway via a positive feedback mechanism contributes to the sustaining activation of PI3K/Akt signaling in ccRCC [178,179]. The rate of genetic alterations in PI3K/Akt pathway components in pRCC is 28% according to the TCGA database, including mutations of *PTEN* and PI3K subunits and amplifications of *GNB2L1*, *PDK1* and *RPTOR* amplifications. In chRCC, *PTEN* was mutated most frequently which occurred in 11% of patients, and mutations of *AKT1*, *TSC1/TSC2* and *mTOR* in the mTOR signaling pathway have also been shown [180].

For targeted therapy towards the mTOR signal pathway in ccRCC, the treatment strategies are at the leading edge, and many drugs have been authorized by the US Food and Drug Administration (FDA). Among these approved drugs, temsirolimus and everolimus are rapalogs that partially inhibit mTORC1 activation, leading to modest survival benefits in advanced ccRCC patients according to the results of the phase III Global ARCC trial [69,181,182]. For metastatic renal cell carcinoma (mRCC), temsirolimus and everolimus are the only mTOR inhibitors authoried by the US FDA. The clinical data demonstrated that mTOR inhibitors can treat mRCC effectively as long as the adverse events were appropriately handled [183]. As reported, ccRCC patients harboring *TSC1* mutation tended to respond to mTOR inhibitors [184]. In a study of 79 patients with mRCC, when treated by mTOR inhibitors, those with mTOR, TSC1, or TSC2 mutations were found to benefit more than others who progressed [185]. Some studies found that resistance to temsirolimus was related to low levels of phosphorylated protein kinase B (p-Akt) and p70 ribosomal S6 Kinase (p-S6K1) in RCC, suggesting that patients with these features should be eliminated from temsirolimus treatments in the future [108]. These data also imply that predicative biomarkers are especially in great need for selecting therapies in future personalized management of RCC [186]. Besides, combination therapy of everolimus together with lenvatinib was regarded to be the first strategy for mRCC, and cabozantinib and nibolumab are subsequent choices, all of which achieved a better efficacy than everolimus alone [187]. mTORC1/2 inhibitors including AZD8055, LN0128 and OSI-027, seem to have potential for greater efficacy than rapalogs in clinical trials of ccRCC [188]. A combination of MAPK- and mTOR-targeted therapies was reported to utilize temsirolimus and tivozanib, which achieved better efficacy in RCC patients [189].

### 3.5. Urinary Bladder Cancer (UBC)

Urinary bladder cancer (UBC), the malignancy that occurs in the urinary system, ranks as the ninth most common cancer [190]. UBC is classified into non-muscle-invasive UBC (NMIUBC) and muscle-invasive UBC (MIUBC) according to the invasion status into the urinary bladder wall and nearby structures. Genetic alterations of the mTOR pathway occur in over 40% of UBC patients, including deletion or mutations of *PTEN*, *TSC1* or *TSC2* and mutations or amplifications of *PI3KCA* or *AKT1* according to the TCGA database [191,192,193,194]. These alterations in the mTOR pathway are reported to be associated with progression and mortality in bladder cancers (*n* = 887) and are valuable for prognosis [195]. UBC patients with a higher grade often harbor mutations that hyperactivate the mTOR pathway or KRAS genes and decrease expression of tumor suppressor genes compared to lower grade UBC patients in whom the FGFR3 mutation dominates [195,196]. Loss of PTEN is common in MIUBC, while is hardly found in NMIUBC [197,198]. In a research composed of both NMIUBC and MIUBC patients, mTOR was expressed in NMIUBC and had a poor prognosis in MIUBC [199]. Another case study (*n* = 208) indicated that mTOR activation evaluated by 4E-BP1 or S6K1 phosphorylation contributed to tumorigenesis and was an indicator of recurrence and poor survival of UBC patients [200].

Research on UBC cell lines 5637, T24, and HT1376 indicated that everolimus and temsirolimus applied as single agent only showed limited efficacy in these experimental trials [201,202]. In ICR mice induced by *N*-butyl-*N*-(4-hydroxybutyl) nitrosamine (BBN), sirolimus decreased tumor incidence and proliferation, as implied by histopathological and immunohistochemical results, while everolimus demonstrated little effects on bladder tumors [203,204]. In addition, sirolimus also showed benefits in a genetically engineered mouse model of invasive UBC [205]. As reported from a phase II study, everolimus demonstrated mild antitumor effects in metastatic UBC patients resistant to chemotherapy [206]. In another phase II study, only a small portion of patients with advanced UBC responded to everolimus [207]. It seems that rapalogs utilized as monotherapy are not as effctive as expected in the treatment of UBC. For combination therapies, the results from 5637 and T24 cell lines were much more exciting because either everolimus or temsirolimus combined with gemcitabine showed a better response, and cisplatin together with everolimus or temsirolimus also achieved a promising results in 5637 and HT1376 cell lines [208]. A synergistic combination of mTOR inhibitors and EGFR/HER2 inhibitors in UBC cell lines implied a potential efficacy in NMIUBC and MIUBC treatments [209]. A study in patient-derived xenograft models with dual inhibition of mTOR and MEK suggested potential clinical efficacy in UBC [210]. Application of mTOR inhibitors in UBC treatments should depend on careful selection of the tumor type: NMIUBC seems to respond to combination of rapalogs and other drugs, while only those MIUBC patients with phosphorylated mTOR are suitable to accept mTOR inhibitors treatments.

### 3.6. Prostate Cancer (PCa)

The mTOR pathway is reported to be significantly active in prostate cancer [211,212]. The PI3K/Akt pathway is found aberrant in PCa cell lines, xenograft models, and 30–50% primary PCa tissue samples [213]. Genetic alterations of the mTOR pathway were detected in 42% of primary prostate tumors and all metastatic tumors [211]. Aberrant PTEN/Akt expression was found in 42% of PCa tissues [214]. As PTEN loss was demonstrated to be associated with a high Gleason score, PCa pathological stages and promoted the progression of lymph node metastasis, PTEN may serve as a potential early prognostic marker in prostate cancers [215,216,217,218,219]. High levels of phosphorylated-4EBP1 and eIF-4E are significantly related to increased mortality in PCa patients, implying that downstream effectors of the mTOR pathway may be a potential prognostic indicator for PCa progression [220]. Studies in PCa cell lines indicated that the PI3K/Akt/mTOR pathway contributed to PCa radioresistance (RR) through mechanisms of intrinsic radioresistance, cancer cell proliferation and hypoxia, and in those PCa RR cell lines, the PI3K/Akt/mTOR pathway was the most active [221,222]. Moreover, activation of the PI3K/Akt/mTOR pathway was also reported to be involved in epithelial mesenchymal transition (EMT) and cancer stem cells (CSCs) in prostate cancer radioresistance [223].

Despite the antitumor efficacy demonstrated by the mTOR inhibitors (rapalogs) rapamycin and everolimus in murine models of Pca [90,224,225], the performances of rapalogs in phase I and II clinical trials were not so satisfactory, leading to application of second generation mTOR inhibitors or further combination therapies in Pca [226,227,228,229]. As reported, the ATP competitive mTOR inhibitor MLN0128 showed better efficacy in reducing tumor size and invasion in cell lines and Pca mouse models [49]. These ATP competitive mTOR inhibitors, such as MLN0128, AZD2014, ZAD8055, CC-223, DS-378a and OSI-027, are in early clinical trials. In preclinical studies, the dual PI3K/mTOR inhibitors BEZ235 and GDC-0980 demonstrated effective inhibition of cell proliferation in prostate cancer cells [230,231]. BEZ235 was also reported to reduce tumor volume in a mouse model harboring PTEN loss, and the effects were enhanced when combined with AR antagonist enzalutamide, implying a potential prospect in synergy treatments cotargeting the AR, PI3K and mTOR signaling pathways in PCa [232]. BEZ235 and GDC-0980 are currently being tested as single agents or combination therapies with abiraterone acetate in the process of phase I/II clinical trials in castration-resistance prostate cancer (CRPC).

### 3.7. Breast Cancer

In breast cancer, most genetic alterations and mutations lie upstream of mTOR resulting in hyperactivation of mTOR signaling. *PIK3CA* is frequently mutated in breast cancer in three “hotspots”: E545K, E542K in exon 9 (helical domain) and H1047R in exon 20 (kinase domain) [233]. As reported, *PIK3CA* mutations occurred in 20–50% of breast cancers, especially including 35% of hormone receptor (HR)-positive breast cancers, 23% of human epidermal growth factor receptor 2 (HER2)-positive breast cancers and less than 10% in triple-negative breast cancer (TNBC) [234]. PTEN mutations occur in less than 3% of breast cancers, while PTEN loss occurs in approximately 30% of breast cancers [234,235]. Although Akt mutations in the catalytic domains have not been detected, E17K substitution occurred in the pleckstrin homology domain of AKT1 resulting in constitutive activation in 3% of HR-positive breast cancers [236]. Studies also found mutations in mTOR itself in various cancer types with FAT and FATC domains frequently mutated [237,238]. Moreover, mTOR expression is correlated with poor prognosis in breast cancer, and phosphor-mTOR was more common in TNBC [239,240,241].

Everolimus has been proved by the FDA in treating hormone receptor-positive, HER2-negative breast cancer. And the mTOR inhibitors have been utilized in many clinical trials in beast cancer treatments, such as HORIZON, BOLERO-1, BOLERO-2, BOLERO-3 and TAMRAD, which are all Phase III or II randomized clinical practices evaluating the combination therapies with different mTOR inhibitors in different settings. The HORIZON trial was executed in first-line patients of Hormone Receptor (HR) positive advanced breast cancer to compare the combined therapy of temsiroliumus with letrozole to therapy of placebo with letrozole. Analysis of the HORIZON trial demonstrated the combination therapy failed to improve PFS and may be account for more grade 3 or 4 adverse effects (37% vs. 24%) [242]. The BOLERO-1 trial was another randomized phase III evaluating everolimus (10 mg) with paclitaxel and trastuzumab in patients of HER2 positive advance breast cancer. PFS was not obviously increased in the group of everolimus (14.9 months) compared to the group of placebo (*P* = 0.1167), while in the HR-negative subgroup, the PFS was prolonged 7.2 months with everolimus administration (*P* = 0.049) [243]. A high rate of adverse events correlated with deaths in everolimus treatments of BOLERO-1 was also reported indicating the necessity to monitor the adverse events in early stage. The object of BOLERO-2 trial is to evaluating combination of mTOR inhibitor everolimus with aromatase inhibitor (AI) in HR positive advanced breast cancers. Application of everolimus increased the PFS to 10.6 months compared to 4.1 months originally with single exemestane administration (*P* < 0.0001) [244], which directly led to the permission of FDA for everolimus with exemestane in advanced breast cancer patients with HR positive and HER2 negative following unsuccessful therapy with letrozole or anastrozole. A recently reported study of BOLERO-2 demonstrated an improvement in overall survival in combination therapy group (31.0 months) compared to the control group with exemestane and placebo (26.6 months) [245]. The TAMRAD trial compared the combination of everolimus with tamoxifen to single tamosifen application in 111 HR positive/HER2 negative, AI resistant metastatic breast cancer patients, implying a significant increase of clinical benefit rate (CBR), time to progression (TTP) and OS by everolimus addition [246]. Analysis of results from HORIZON and BOLERO-2 illuminated that endocrine-resistant patients may gain more benefits from temsirolimus administration. So far, researches have mainly focused on clinical efficacy in HR positive and HER2 negative breast cancer patients, in which everolimus has been approved for combined application with exemestane. Ridaforolimus was reported to benefit HER2 positive metastatic breast cancer patients when applied with trastuzumab in a phase II trial, indicating ridaforolimus may improve the efficacy of trastuzumab [247].

Aside from the rapalogs, other mTOR inhibitors, such as ATP competitive inhibitors and PI3K /mTOR dual inhibitors, have also been studied in breast cancer. ATP competitive inhibitors, AZD2014, which showed better anti-proliferative capabilities in breast cancer cell lines, xenograft and primary explant models, is now in process of phase II clinical trials designed to be combined with other compounds or therapies [112,248,249]. MLN0128 inhibited cell viability in five breast cell lines (HR−/+, HER2−/+) and acted synergistically with TSA [250]. In a phase I trial, CC223 was reported to be tolerated well and achieved partial response in breast cancer patients, implying its promising potential in the future [251]. Dual inhibitors of PI3K and mTOR, BEZ235 and PF-04691502, both demonstrated antitumor efficacy in breast cancer cells and xenograft models [252,253], but were also inclined to cause serious side effects in clinical practices. More combination therapies with mTOR inhibitors are still underway in different settings [254].

### 3.8. Head and Neck Squamous Cell Carcinoma (HNSCC)

Head and neck squamous cell carcinoma (HNSCC) accounts for almost 90% of human head and neck cancers, including cancers in the oral cavity, oropharynx, nasopharynx, hypopharynx, and larynx.

A whole-exome sequencing research in 151 HNSCC patients demonstrated that PI3K pathway was frequently mutated in 30.5% of HNSCC [255]. The genes with genetic alterations in HNSCC mainly include *PIK3CA*, *PIK3CD*, *PTEN*, *PDK1*, *Akt*, *RICTOR*, *RAPTOR*, *TSC1*, *TSC2* and *mTOR* [256,257,258,259,260]. Especially, *PI3KCA* amplifications and *PTEN* mutation are prevalent in human papilloma virus (HPV) infected HNSCC [261]. Another separate study indicated that HPV positive HNSCC had a different mutated gene cluster from HPV negative HNSCC [262]. PI3KCA amplification was observed in early stage in the carcinogenesis as well as in the malignancies, implying PI3K pathway contributes to the oncogenic process of HNSCC [263]. In addition, advanced HNSCC patients often harbor multiple aberrations including mutations in *PIK3CA* and *mTOR* or *PIK3CA* and *PTEN*, suggesting these simultaneously existing mutations are also associated with HNSCC progression [264]. A phase II clinical trial showed that the single-nucleotide polymorphisms (SNP) in PTEN (rs12569998) and AKT2 (rs8100018) are related with the progression risk and PFS in metastatic HNSCC treated with combination of docetaxel and cetuximab [265]. mTOR is reported to be activated in 80–90% HNSCC, particularly those with HPV infection [266,267]. As reported, mTOR and its downstream effectors, eIF-4E, 4EBP1, S6K1, and S6 are all biomarkers for diagnosis and prognosis in head and neck cancer, demonstrating the promising prospect for mTOR inhibitors in HNSCC treatments [268].

In preclinical studies of mTOR inhibitors, rapamycin and its rapalog temsirolimus, everolimus all showed efficacy in xenograft HNSCC models [268,269]. An in vivo retroinhibition approach applied in HNSCC cells demonstrated that rapamycin and its rapalogs can prevent angiogenesis, and another study in xenograft model implied that rapamycin and rapalog everolimus also inhibit lymphangiogenesis and lymph node metastasis in HNSCC [270,271]. Besides, mTOR inhibition can also act synergistically with radiation therapy to reinforce the anti-angiogenic effects and suppress HNSCC tumor growth in xenograft models [272,273]. Besides, several reports demonstrated the promising results of mTOR inhibitors in HNSCC patient-derived tumorgraft (PDX) models [274,275,276].

Rapamycin, originally regarded as a specific inhibitor of mTORC1, was found to supress both mTORC1 and mTORC2 in HNSCC cells [267]. And in a study of newly diagnosed HNSCC patients, rapamycin (NCT01995922) achieved improved effectiveness, as most patients responded and one patient got complete response [260]. Everolimus has been utilized in combination with cisplatin and radiation therapy or with erlotinib or with cisplatin and docetaxel in HNSCC treatment, and was tolerated well in these phase I or II clinical trials [277,278,279]. Another combination therapy in a phase I study with temsirolimus, carboplatin and paclitaxel in HNSCC achieved a partial response rate of 22%, while temsirolimus combined with erlotinib was poorly tolerated with common adverse effects including fatigue, hyperglycemia, diarrhea and peritonitis in recurrent or metastatic HNSCC patients in a phase II strudy [280,281]. Actually, most clinical practices of mTOR inhibitors as single agent in HNSCC have been applied in those patients that failed in other therapies or general patients without selection. Clinical trails focusing on mTOR inhibitors in HPV+ HNSCC patients have seldom been conducted yet, although previous researches confirmed the potential of this strategy. Dual PI3K/mTOR inhibitors like BEZ235 showed anti-tumor effects in HNSCC cell lines and tumorgrafts with PIK3CA mutations, its efficacy in HNSCC patients remained unknown [255]. Besides, combination therapies of mTOR inhibitors with other molecular-targeted therapies (EGFR, VEGFR, MEK, MAPK and MET) or conventional therapies may shed lights in HNSCC clinical success.

## 4. Discussions and Future Prospects

Among these eight solid tumor types we discussed, it seems that mTOR inhibitors achieved better efficacy and relatively more attentions in treatments of renal cancer and breast cancer. Although these tumors originate from different primary organs, they share similar genetic alterations in PI3K or mTOR signal pathway (as summarized in Table 1), which imply that genetical and molecular biological methods should be applied to classify cancer subtypes in addition to those organs affected especially before targeted therapy application. Then we can get some related clues from clinical trials about which specific mTOR inhibitors or combinations may benefit cancer patients with what kind of genetic alterations in mTOR signaling [112]. We also summarize mTOR inhibitors that are under preclinical and clinical trials in these eight solid cancer types (as shown in Table 2). Apart from those eight types of solid tumors we mentioned, mTOR inhibitors have also been utilized in the therapies of gynecologic cancer, osteosarcoma, leukemia, lymphoma, thyroid carcinoma, glioblastoma, neuroendocrine tumors and medulloblastoma, and we won’t go into details here [282,283,284,285,286,287,288,289].

To summarize, mTOR inhibitors can be classified into three generations: the first generation inhibitors, mainly include rapamycin and its rapalogs temsirolimus (CCI-779), everolimus (RAD001) and ridaforolimus; second generation inhibitors refer to ATP-competitive inhibitor of mTOR kinase which inhibit both mTORC1 and mTORC2 simutaneously (MLN0128, AZD2014, AZD8055, CC223, etc.) as well as some dual PI3K/mTOR inhibitors (PP242, MLN0128, KU-0063794, BEZ235, etc.); Third generation inhibitor, which has been seldom reported in clinical trials yet, has a bivalent structure to take advantage of the two docking sites and avoid resistance against the original compounds. Better efficacy with less toxicity in large individual variability is always the ultimate aim for designing targeting drugs. Rapalogs, as the first generation mTOR inhibitors, have been tested in many clinical trials, but they achieved only modest efficacy applied as monotherapies in cancer treatments due to multiple mechanisms: First, rapalogs partially inhibit mTORC1 activity, and a negative feed back loop will arouse the PI3K and Akt signal via PI3K/mTORC2/Akt cascade, leading to increased cell growth and enhanced cell survival [290,291]. mTOR signal pathway is a complicated system which has various cross-talks with other signaling pathways that can counteract rapalogs’ functions [292]. Second, although phosphorylation of S6K1 is totally blocked by rapalogs, 4EBP1 phosphorylation is modestly suppressed. Thus, proteins translation regulated by 4EBP1 in tumorigenesis can still be translated to promote cancer progression. Also, rapalogs decrease the inhibition of IRS-1 by S6K1 phosphorylation, inducing Akt signaling and downstream pathways [291]. Besides, mTORC1 inhibition can also promote cell proliferation by catabolism of extracellular proteins in nutrient deprived conditions, and enhance cell survival via autophagy [293,294]. Therefore, new focuses are turned to the second generation mTOR inhibitors with dual inhibition on PI3K and mTOR signaling or mTOR kinase inhibitors, which are less possible to induce drug resistance than rapalogs alone and already have been introduced in preclinical study or entered the clinical practices [170]. Combination therapies with rapalogs and other signal pathway inhibitors as well as conventional therapies are more prosperous, and many clinical trials have already confirmed the benefits of this treating strategy in various cancer types as we discussed above. However, whether these therapy strategies will offer improved benefits need to be verified in further clinical trials.

For future directions of mTOR targeting therapy, we should clarify the following issues: first, we need to establish appropriate dose schedules of mTOR inhibitors that ensure the efficacy and better toleration in patients; second, all the mTOR inhibitors related treatments no matter monotherapies or combination therapies should continue to be carefully optimized and evaluated to achieve the best effectiveness in clinical trials; third, we should improve the ability to predict who will respond to a certain targeted therapy of mTOR according to the analysis of genetic variations from the patients; fourth, molecular biomarkers for the prognosis and prediction need to be explored to help selecting suitable therapy plans and monitoring the treatment response to mTOR inhibitors in patients.

In the present review, we discuss the mTOR components of mTORC1 and mTORC2 and the upstream and downstream effectors of mTOR signaling pathway in physiological and pathological status. Genetic alterations occurred in eight solid tumors and preclinical as well as clinical trials targeting mTOR in these tumor types. As we know, most tumors are heterogeneous and caused by multiple genetic and environmental factors, so it is difficult to have one single drug to fit all patients with the same tumor type. More thorough realization of genetic profile and molecular characterization of different cancer subtypes will surely help us select the most appropriate drugs in targeting mTOR signaling in cancer therapy. With the rapid development of biomarkers and deep sequencing technology, personalized therapy utilizing more specific mTOR targeting drugs that have better efficacy and more safety, will be translated into clinical cancer treatments in the near future.

## Figures and Tables

**Table 1:** The incidence of genetic variations in mTOR (The mammalian or mechanistic target of rapamycin) signal pathway components in 8 types of solid human cancers summarized in this review.

Cancer Type	Refs	Type of Genetic Variation	Gene (Incidence)
**Lung cancer**			
Squamous cancer	[114]	genetic alterations	*PI3CA* (16%), *PTEN* (15%), *AKT3* (16%), *AKT2* (4%), AKT1(<1%)
		amplifications	*PI3CA* (33%)
SCLC	[115]	genetic alterations	*PI3CA* (6%), *PTEN* (4%), *AKT3* (4%), *AKT2* (9%), *RICTOR* (9%), *mTOR* (4%)
**Gastric cancer**			
	[131,132]	mutations	*PI3CA* (18%) (E545K, E542K-exon9, H1047R-exon20)
		amplifications	*PI3CA* (5%)
	TCGA	deletions, mutations, amplifications	*PTEN* (0.3%, 3.1%, 4% )
	[135]	deletions and mutations	*PTEN* (19%, Chinese population
	[137,138]	genetic alterations	*AKT* (1–3%)
**Colorectal cancer**			
	[154,155]	mutations	*PI3CA* (15%)
		deletions	*PTEN* (20–40%)
**Renal cancer**			
ccRcc	[70,176,177]	amplifications	*GNB2L1* (6%)
		amplifications or mutations	*PI3KCA* (5%)
		deletions or mutations	*PTEN* (5%)
		mutations	*mTOR* (6%)
pRcc	TCGA	mutations	*PTEN*, *PI3K*
		amplifications	*GNB2L1*, *PDK1*, *RPTOR* ( total: 28%)
chRCC	[180]	mutations	*PTEN* (11%)
**Urinary bladder cancer**			
	[192]	activating point mutations	*PI3KCA* (17%)
		mutations or deletions	*TSC1* or *TSC2* (9%)
		mutations	*AKT3* (10%)
**Prostate cancer**			
	[211]	genetic alterations	mTOR pathway (42% primary PCa, 100% metastatic PCa)
		mutations	*PTEN* (4% primary PCa, 42% metastatic PCa); *PIK3CA* (6% primary PCa, 16% metastatic PCa)
**Breast cancer**			
	[233,234,235]	mutations	*PIK3CA* (20–50%) (E545K, E542K-exon9, H1047R-exon20)
		mutations, loss	*PTEN* (<3%, 30%)
HR-positive	[234]	mutations	*PIK3CA* (35%)
	[236]	E17K substitution	*AKT1* (3%)
HER2-positive	[234]	mutations	*PIK3CA* (23%)
TNBC	[234]	mutations	*PIK3CA* (<10%)
**Head and neck squamous cell carcinoma**			
	[255,257,258,260]	mutations	*PIK3CA* (12.6%, 11–40%) (E545K, E542K-exon9, H1047R-exon20)
			*TSC1* (11%), *TSC2* (13%)
		amplifications	*PIK3CA* (24.4% )
		loss	*PTEN* (8.16%, 10–15%)

**Table 2:** mTOR inhibitors that are under preclinical and clinical trials in eight solid cancer types summerized in this review.

Cancer Type	Drug Class	Drugs	Refs
**Lung cancer**			
NSCLC	mTORC1 inhibitors	everolimus	[118,119,120,121,122,123,124]
		temsirolimus	[125,126,127]
		sirolimus	[128]
**Gastric cancer**			
	mTORC1 inhibitors	rapamycin	[145,146]
		temsirolimus	[126]
		everolimus	[143,144,148,149,150]
		ridaforolimus	[151]
	mTORC1 and mTORC2 inhibitors	PP242	[138,152]
**Colorectal cancer**			
	mTORC1 inhibitor	temsirolimus	[162,163]
		rapamycin	[164,166]
		everolimus	[165,167,168]
	PI3K and mTOR inhibitors	NVP-BEZ235	[171,174]
	mTORC1 and mTORC2 inhibitors	OSI-027	[172,173]
		PP242	[174]
**Renal cancer**			
ccRCC	mTORC1 inhibitor	temsirolimus	[108,181,189]
		everolimus	[182,187]
	mTORC1 and mTORC2 inhibitors	AZD8055, IN-0128, OSI-027	[188]
mRCC		rapamycin	[184,185]
**Urinary bladder cancer**			
	mTORC1 inhibitor	rapamycin	[201,205]
		everolimus	[202,204,206,207,208]
		sirolimus	[203,205]
		temsirolimus	[208]
	mTORC1 and mTORC2 inhibitors	PP242 or OSI-027	[209]
**Prostate cancer**			
	mTORC1 inhibitor	rapamycin	[225,227]
		everolimus	[226,228,229]
	mTORC1 and mTORC2 inhibitors	MLN0128	[49]
	PI3K and mTOR inhibitors	NVP-BEZ235, GDC-0980	[230,231]
**Breast cancer**			
	mTORC1 inhibitor	rapamycin	[242]
		everolimus	[243,244,245,246]
		ridaforolimus	[247]
	mTORC1 and mTORC2 inhibitors	AZD2014	[112,248,249]
		MLN0128	[250]
		CC-223	[251]
	PI3K and mTOR inhibitors	PF-04691502	[252]
		NVP-BEZ235	[253]
**Head and neck squamous cell carcinoma**			
	mTORC1 inhibitor	rapamycin	[267,268,269,270,271]
		temsirolimus	[268,273,280,281]
		everolimus	[268,270,274,277,278,279]
	PI3K and mTOR inhibitors	PF-05212384	[272]

## Abbreviations

4EBP1	Eukaryotic translation initiation factor 4E binding protein 1
AEs	Adverse events
AMPK	AMP kinase
AMPKβ	AMP activated protein kinaseβ
ATP	Adenosine Tri-Phosphate
BBN	N-butyl-N-(4-hydroxybutyl) nitrosamine
Bgff	Basic fibroblast growth factor
CBR	Clinical benefit rate
ccRCC	Clear cell renal cell carcinoma
chRCC	Chromophobe renal cell carcinoma
CRC	Colorectal cancer
CRPC	Castration-resistance prostate cancer
CSC	Cancer stem cells
CT	Chemotherapy
DAP1	Death-associated protein 1
DCR	Disease control rate
DEP	EGL-10 and Pleckstrin
DEPTOR	DEP domain-containing mTOR-interacting protein
EBV	Epstein-Barr virus
EGFR	Epidermal Growth Factor Receptor
eIF-3	Eukaryotic initiation factor-3
eIF-4E	Eukaryotic translation Initiation Factor
EMT	Epithelial mesenchymal transition
ERKs	Extracellular signal-regulated kinases
FDA	Food and Drug Administration
FKBP12	FK506 Binding Protein 12
FoxO	Forkhead box family transcription factors
GC	Gastric cancer
Grb10	Growth factor receptor-bound protein 10
HER2	Human epidermal growth factor receptor 2
HIF-1α	Hypoxia-inducible factor 1α
HNSCC	Head and neck squamous cell carcinoma
HPV	Papilloma virus
HR	Hormone Receptor
IGFR	Insulin growth factor receptor
IRS	Insulin receptor substrate
LAR	Long-acting release
MAPK	Mitogen activated protein kinase
MEK	Mitogen-activated protein kinase kinase
MIUBCs	Muscle-invasive UBCs
mLST8	Mammalian lethal with SEC13 protein 8
mRCC	Metastatic renal cell carcinoma
mSIN1	Mammalian stress-activated protein kinase interacting protein 1
mTOR	The mammalian or mechanistic target of rapamycin
mTORC	mTOR complex
NDRG1	N-myc Downstream-Regulated Gene 1 protein
NF-κB	Nuclear factor-κB
NMIUBCs	Non-muscle-invasive UBCs
NSCLC	Non-small cell lung cancer
OS	Overall survival
p-Akt	Phosphor-protein kinase B
PCa	Prostate cancer
PDGF	Platelet-derived growth factor
PDK1	Phosphoinositide-dependent Kinase 1
PFS	Progression free survival
PI3K	Phosphoinositide 3-kinase
PIKK	Phosphoinositide 3-kinase related protein kinase
PIP3	Phosphatidylinositol (3, 4, 5)-triphosphate
PKA	cAMP-dependent protein kinase
PKC	Protein kinase C
PKG	cGMP-dependent protein kinase
p-mTOR	Phosphorylated-mTOR
PP2A	Protein phosphatase 2A
PRAS40	Proline-rich substrate of 40 kDa
PRCC	Papillary renal cell carcinoma
Protor-1/2	Protein observed with RICTOR 1/2
PRR 5	Proline-rich protein 5
p-S6K1	p70 ribosomal S6 Kinase
PTEN	Phosphatase and tensin homolog on chromosome 10
RAPTOR	Regulatory-associated protein of mTOR
RCC	Renal cancer
REDD1	DNA damage and development 1
RHEB	Ras homolog enriched in brain
RICTOR	Rapamycin-insensitive companion of mTOR
RR	Radioresistance
RSK	Ribosomal protein S6 kinase
S6K	S6 kinase
S6RP	S6 ribosomal protein
SCLC	Small cell lung cancinoma
SGK	Serum glucose kinase
SNP	Single-nucleotide polymorphisms
SQCLC	Squamous cell lung carcinoma
SREBP	Sterol regulatory element-binding protein
SRE-BP1	Sterol regulatory element-binding protein 1
STAT	Signal transducer and activator of transcription
STK11	Serine threonine kinase 11
TCGA	The cancer genome atlas
Tel2	Telomere maintenance 2
TFEB	Transcription factor transcription factor EB
TNBC	Triple-negative breast cancer
TSC	Tuberous sclerosis complex
Tti1	Tel2-interacting protein 1
TTP	Time to progression
UBC	Urinary bladder cancer
ULK1	UNC-5 Like autophagy activating Kinase 1
VEGF	Vascular endothelial growth factor
WIPI2	WD repeat domain phophoinositide-interacting protein 2
